# Stress-Strength Parameter Estimation under Small Sample Size: A Testing Hypothesis Approach

**DOI:** 10.1155/2021/8705547

**Published:** 2021-09-30

**Authors:** Hassan Alsuhabi, Mohammad Mehdi Saber, M. M. Abd El-Raouf

**Affiliations:** ^1^Department of Mathematics, Al-Qunfudah University College, Umm Al-Qura University, Mecca, Saudi Arabia; ^2^Department of Statistics, Higher Education Center of Eghlid, Eghlid, Iran; ^3^Basic and Applied Science Institute, Arab Academy for Science, Technology and Maritime Transport (AASTMT), Alexandia, Egypt

## Abstract

In this paper, uniformly most powerful unbiased test for testing the stress-strength model has been presented for the first time. The end of the paper is recommending a method which is appropriate for no large data where a normal asymptotic distribution is not applicable. The previous methods for inference on stress-strength models use almost all the asymptotic properties of maximum likelihood estimators. The distribution of components is considered exponential and generalized logistic. A corresponding unbiased confidence interval is constructed, too. We compare presented methodology with previous methods and show the method of this paper is logically better than other methods. Interesting result is that our recommended method not only uses from small sample size but also has better result than other ones.

## 1. Introduction

In reliability literature, the quantity *R*=*P*(*X* > *Y*) is often referred to the stress-strength model. In addition to reliability, this parameter has application in some scientific fields such as biostatistics, quality control, engineering, stochastic precedence, and probabilistic mechanical design. Kotz et al. [1] and Ventura and Racugno [2] have presented a comprehensive review on this matter, especially from its applications. Regarding [1] an instance of real practice of the stress-strength model is in a clinical study, where *Y* and *X* are assumed as the outcomes of a treatment and a control group, respectively. Then, the ineffectiveness of the treatment is measured by *R*. In terms of reliability, *Y* is considered the strength of a component, which is under *X* stress. Henceforth, two quantities *R* and (1 − *R*) indicate the probabilities of system performance and system failure, respectively. Many distributions have been applied by authors for estimation of *R*. For instance, see Rezaei et al. [3] and Nadar et al. [4] for a nearly complete list of distributions used in this matter.

A distribution, whose application in reliability and especially in estimation of *R*_ _ has established, is generalized logistic (GL) distribution. This distribution, as one of three generalized forms of the standard logistic distribution, has been defined by Balakrishnan and Leung [5]. A random variable *X* is said to have a GL distribution if it has the following probability density function:

(1) $$\begin{array}{c} f\left(x\right)=\alpha \lambda \;e^{-\lambda x}\;{\left(1+e^{-\lambda x}\right)}^{-\alpha -1},\;\alpha >0,\;\lambda >0,\;x\in R, \end{array}$$

which is denoted by GL(*α*, *λ*). Furthermore, its cumulative distribution function is

(2) $$\begin{array}{c} F\left(x\right)={\left(1+e^{-\lambda x}\right)}^{-\alpha }. \end{array}$$

Here, *α* and *λ* are the shape and scale parameters, respectively.

It has also been called the skew-logistic distribution and is defined on (−*∞*, *∞*). Estimation of GL distribution parameters has been received attention for practical usage by some works such as Balakrishnan [6], Asgharzadeh [7], and Gupta and Kundu [8]. Among the recent articles, Alkasasbeh and Raqab [9] estimated the unknown parameters of the GL lifetime model using different approaches and Vasudeva Rao et al. [10] obtained maximum likelihood estimation by a linear approximation. In stress-strength literature, when distribution of components is GL, Asgharzadeh et al. [11] and Rasekhi et al. [12] have investigated in statistical inference of *R* and its multicomponent version, respectively.

In almost all studies, authors have applied maximum likelihood estimator (MLE) and its asymptotic distribution for estimating and inference on *R*. This method leads to asymptotic Confidence Interval (C.I) for *R* and analogously asymptotic Critical Region (C.R) for testing hypotheses on *R*. These asymptotic C.I and C.R have suitable efficiency for data with large sample size. In the other words, performance of any asymptotic method for small data is not appropriate. For small sample size, nonasymptotic and exact statistical methods are needed in order to reach reliable results. However, an exact C.I and C.R of *R* has not been presented in previous works. Therefore, we are motivated for estimation of *R* with small sample size based on a testing hypothesis approach and finding its Uniformly Most Powerful Unbiased Test (UMPUT). The mentioned methodology of this study has been applied for the first time in stress-strength literature. For this goal, we find UMPUT and its corresponding C.I for *R* in the case where components have exponential distribution. Then, by using of relationship between exponential distribution and GL distribution, our findings are applied and modified to the case of GL distributed components. We have chosen GL distribution since, by its relation with exponential distribution paper, it covers this distribution, too. On the contrary, GL distribution has been used in many other previous papers on stress-strength models, which is mentioned above. Also, GL distribution is flexible and can be fitted to numerous datasets.

The rest of the paper is organized as follows. We obtain UMPUT and unbiased C.I for *R* when components have exponential distributions in Section 2.  Section 3 is devoted to similar work as Section 2, however, for GL distributed components. A comparison between the introduced method and previous methods which are based on asymptotic distribution of maximum likelihood estimators has been provided in Section 4.

## 2. UMPUT for *R* in Exponential Distribution

Suppose random variables *X* and *Y* are independent and *X* ~ *E*(*λ*_1_) and *Y* ~ *E*(*λ*_2_); then, *R*=*P*(*X* > *Y*)=(*λ*_2_/*λ*_1_+*λ*_2_). Throughout this section, this supposes that *X*_1_,…, *X*_*n*_ ~ *X* and *Y*_1_,…, *Y*_*m*_ ~ *Y*. An asymptotic C.I for *R* has been obtained in the following proposition.

**Proposition 1 .**

The ML estimators for parameters are ${\hat{\lambda }}_{1}=\left(1/\bar{X}\right)$ and ${\hat{\lambda }}_{2}=\left(1/\bar{Y}\right)$, so $\hat{R}=\left({\hat{\lambda }}_{2}/{\hat{\lambda }}_{1}+{\hat{\lambda }}_{2}\right)$. Also, $\left(\hat{R}-R/{\hat{\sigma }}_{1}\right)⟶N\left(0,1\right)$ as *n* and *m* tend to infinity and (*n*/*m*)⟶*d*, for 0 < *d* < +*∞*, where ${\hat{\sigma }}_{1}^{2}=\left(n+m/nm\right)\left({\hat{\lambda }}_{1}^{2}{\hat{\lambda }}_{2}^{2}/{\left({\hat{\lambda }}_{1}+{\hat{\lambda }}_{2}\right)}^{4}\right)$.

From this proposition, (1 − *α*) × 100 percentage C.I of *R* is given by

(3) $$\begin{array}{c} R\in \left(\hat{R}-z_{1-\left(\alpha /2\right)}\;{\hat{\sigma }}_{1}\;,\;\hat{R}+z_{1-\left(\alpha /2\right)}\;{\hat{\sigma }}_{1}\right). \end{array}$$

We define three testing hypotheses about *R* as $\left\{\begin{array}{c} H_{0}:R=r\\ K_{0}:R>r \end{array}\right.$, $\left\{\begin{array}{c} H_{0}:R=r\\ K_{1}:R<r \end{array}\right.$, and $\left\{\begin{array}{c} H_{0}:R=r\\ K_{2}:R\neq r \end{array}\right.$. The critical region (C.R) for these tests based on a normal approximation are C.R_*as*,0_={*Z* > *z*_1−*α*_}, C.R_*as*,1_={*Z* < *z*_*α*_}, and C.R_*as*,2_={|*Z*| > *z*_1−(*α*/2)_}, respectively where $Z=\left(\hat{R}-r/{\hat{\sigma }}_{1}\right)$.

In continuation of this section, we find UMPUT for above tests. First, we consider problem of comparing parameters of two independent exponential populations in Theorem 1.

**Theorem 1 .**

Consider problem of testing hypotheses $\left\{\begin{array}{c} H_{E,0}:\lambda_{2}=\lambda_{1}\\ K_{E,0}:\lambda_{2}>\lambda_{1} \end{array}\right.$, $\left\{\begin{array}{c} H_{E,0}:\lambda_{2}=\lambda_{1}\\ K_{E,1}:\lambda_{2}<\lambda_{1} \end{array}\right.$, and $\left\{\begin{array}{c} H_{E,0}:\lambda_{2}=\lambda_{1}\\ K_{E,2}:\lambda_{2}\neq \lambda_{1} \end{array}\right.$. Then,

(i) C.Rs of UMPUTs for *K*_*E*,0_ and *K*_*E*,1_ are given by C.R_UMPU,0_={*W* > *q*beta(1 − *α*, *n*, *m*)} and C.R_UMPU,1_={*W* < *q*beta(*α*, *n*, *m*)}, respectively. Here, *q*beta(*α*, *n*, *m*) is *α*th quantile of distribution beta(*n*, *m*).
(ii) Also, the acceptance region of UMPUT for *K*_*E*,2_ is A.R_UMPU,2_={*d*_*α*_ < *W* < *c*_*α*_}, which *d*_*α*_ and *c*_*α*_ are determined by the following equations:
  (4) $$\begin{array}{c} p\text{beta}\left(c_{\alpha },n,m\right)-p\text{beta}\left(d_{\alpha },n,m\right)=1-\alpha , \end{array}$$
  (5) $$\begin{array}{c} p\text{beta}\left(\frac{c_{\alpha }}{T},n+1,m\right)-p\text{beta}\left(\frac{d_{\alpha }}{T},n+1,m\right)=\left(1-\alpha \right)p\text{beta}\left(\frac{1}{T},n+1,m\right), \end{array}$$
where *W*=(*S*/*T*), *S*=∑_*i*=1_^*n*^*X*_*i*_, *T*=∑_*i*=1_^*n*^*X*_*i*_+∑_*j*=1_^*m*^*Y*_*j*_, and *p*beta(*b*, *n*, *m*) is cumulative distribution function of distribution beta(*n*, *m*) in *b*.

**Proof**

First, notice that if random variable *B* has a beta distribution *B* ~ beta(*n*, *m*), then *P*(*B* < *q*beta(*α*, *n*, *m*))=*α* and *P*(*B* < *b*)=*p*beta(*b*, *n*, *m*).

The joint distribution of *X*=(*X*_1_,…, *X*_*n*_) and *Y*=(*Y*_1_,…, *Y*_*m*_) is given by

(6) $$\begin{array}{c} f_{X,Y}\left(x,y\right)=\lambda_{1}^{n}\lambda_{2}^{m}e^{-\lambda_{1}\sum_{i=1}^{n}x_{i}-\lambda_{2}\sum_{j=1}^{m}y_{j}}⋉e^{\left(\lambda_{2}-\lambda_{1}\right)\sum_{i=1}^{n}x_{i}-\lambda_{2}\left(\sum_{j=1}^{m}y_{j}+\sum_{i=1}^{n}x_{i}\right)}. \end{array}$$

Therefore, regarding Lehmann and Romano [13], in Section 3, there exists UMPUT of three hypotheses concerning the parameter *θ*=*λ*_2_ − *λ*_1_. The tests are performed based on *S*=∑_*i*=1_^*n*^*X*_*i*_ conditionally on *T*=∑_*i*=1_^*n*^*X*_*i*_+∑_*j*=1_^*m*^*Y*_*j*_. Our computations show that the conditional distribution of *S* given *T* is as follows:

(7) $$\begin{array}{c} f_{S|T}\left(s,t\right)=\frac{\Gamma \left(n+m\right)}{\Gamma \left(n\right)\Gamma \left(m\right)}\frac{1}{t}{\left(\frac{s}{t}\right)}^{n-1}{\left(1-\frac{s}{t}\right)}^{m-1}. \end{array}$$

In fact, *S|*(*T*=*t*) ~ *t*beta(*n*, *m*).

Now, C.R of *K*_*E*,0_ is given by C.R_UMPU,0_={*S* > *c*_*α*,*t*_} and ∫_0_^*c*_*α*,*t*_^*f*_*S|T*_(*s*, *t*) d*s*=1 − *α*. Substitution of equation (7) in the above integral and using a change of variable *z*=(*S*/*t*) leads to

(8) $$\begin{array}{c} \int_{0}^{\left(c_{\alpha ,t}/t\right)}\frac{\Gamma \left(n+m\right)}{\Gamma \left(n\right)\Gamma \left(m\right)}z^{n-1}{\left(1-z\right)}^{m-1}\text{ d}z=1-\alpha , \end{array}$$

which shows *c*_*α*,*t*_=*tq*beta(1 − *α*, *n*, *m*). Therefore, C.R_UMPU,0_={*S* > *Tq*beta(1 − *α*, *n*, *m*)} completes the first part of proof. The CR of *K*_*E*,1_ named C.R_UMPU,1_ is earned by similar computations.

For proof of part (ii), we have C.R_UMPU,2_={*S* < *d*_*α*,*t*_ or *S* > *c*_*α*,*t*_}, where *d*_*α*,*t*_ and *c*_*α*,*t*_ are determined by

(9) $$\begin{array}{c} E\left(I_{\text{C}{\text{.R}}_{\text{UMPU},2}}|t\right)=\alpha \end{array}$$

and

(10) $$\begin{array}{c} E\left(S\;I_{\text{C}{\text{.R}}_{\text{UMPU},2}}|t\right)=\alpha \;E\left(\;S|t\right). \end{array}$$

From equation (9), we have ∫_*d*_*α*,*t*__^*c*_*α*,*t*_^*f*_*S|T*_(*s*, *t*) d*s*=1 − *α* which by thw similar method with (i) leads to equation (4).

Solving equation (10) is the last pace of proof, first, we compute *E*(*S|t*) and *E*(*SI*_C.R_UMPU,3__*|t*) as follows:

(11) $$\begin{array}{c} E\left(\;S|t\right)=\int_{0}^{1}s\;f_{S|T}\left(s,t\right)\text{ d}s=t\int_{0}^{\left(1/t\right)}\frac{\Gamma \left(n+m\right)}{\Gamma \left(n\right)\Gamma \left(m\right)}\;z^{n}{\left(1-z\right)}^{m-1}\text{ d}z=\frac{n}{n+m}tp\text{beta}\left(\frac{1}{t},n+1,m\right),\\ E\left(SI_{\text{C}{\text{.R}}_{\text{UMPU},3}}\;|t\right)=t\left\{\int_{0}^{\left(d_{\alpha ,t}/t\right)}\frac{\Gamma \left(n+m\right)}{\Gamma \left(n\right)\Gamma \left(m\right)}\;z^{n}{\left(1-z\right)}^{m-1}\text{ d}z+\int_{\left(c_{\alpha ,t}/t\right)}^{\left(1/t\right)}\frac{\Gamma \left(n+m\right)}{\Gamma \left(n\right)\Gamma \left(m\right)}\;z^{n}{\left(1-z\right)}^{m-1}\text{ d}z\right\}\\ =t\left\{\int_{0}^{\left(1/t\right)}\frac{\Gamma \left(n+m\right)}{\Gamma \left(n\right)\Gamma \left(m\right)}\;z^{n}{\left(1-z\right)}^{m-1}\text{ d}z-\int_{\left(d_{\alpha ,t}/t\right)}^{\left(c_{\alpha ,t}/t\right)}\frac{\Gamma \left(n+m\right)}{\Gamma \left(n\right)\Gamma \left(m\right)}\;z^{n}{\left(1-z\right)}^{m-1}\text{ d}z\right\}. \end{array}$$

From these equations, we have

(12) $$\begin{array}{c} \alpha p\text{beta}\left(\frac{1}{t},n+1,m\right)=p\text{beta}\left(\frac{1}{t},n+1,m\right)-\int_{\left(d_{\alpha ,t}/t\right)}^{\left(c_{\alpha ,t}/t\right)}\frac{\Gamma \left(n+m\right)}{\Gamma \left(n\right)\Gamma \left(m\right)}\;z^{n}{\left(1-z\right)}^{m-1}\text{ d}z, \end{array}$$

which clearly leads to equation (5).

Now, we can apply Theorem 1 in order to find UMPUTs of hypotheses *K*_0_, *K*_1_, and *K*_2_.

**Theorem 2 .**

The CRs of UMPUTs for *K*_0_ and *K*_1_ are

(13) $$\begin{array}{c} \text{C}{\text{.R}}_{\text{UMPU},0*}=\left\{W^{*}>q\text{beta}\left(1-\alpha ,n,m\right)\right\} \end{array}$$

and

(14) $$\begin{array}{c} \text{C}{\text{.R}}_{\text{UMPU},1*}=\left\{W^{*}<q\text{beta}\left(\alpha ,n,m\right)\right\}, \end{array}$$

respectively.

Also, the acceptance region of UMPUT for *K*_2_ is

(15) $$\begin{array}{c} \text{A}{\text{.R}}_{\text{UMPU},2*}=\left\{d_{\alpha }<W^{*}<c_{\alpha }\right\}, \end{array}$$

In which *d*_*α*_ and *c*_*α*_ are determined by the following equations:

(16) $$\begin{array}{c} p\text{beta}\left(c_{\alpha },n,m\right)-p\text{beta}\left(d_{\alpha },n,m\right)=1-\alpha , \end{array}$$

(17) $$\begin{array}{c} p\text{beta}\left(\frac{c_{\alpha }}{T^{*}},n+1,m\right)-p\text{beta}\left(\frac{d_{\alpha }}{T^{*}},n+1,m\right)=\left(1-\alpha \right)p\text{beta}\left(\frac{1}{T^{*}},n+1,m\right), \end{array}$$

where *S*^*∗*^=(1 − *r*/*r*)∑_*i*=1_^*n*^*X*_*i*_, *T*^*∗*^=(1 − *r*/*r*)∑_*i*=1_^*n*^*X*_*i*_+∑_*j*=1_^*m*^*Y*_*j*_, and *W*^*∗*^=(*S*^*∗*^/*S*^*∗*^+*T*^*∗*^).

**Proof**

Notice that *R*=(*λ*_2_/*λ*_1_+*λ*_2_)=*r* is equivalent with *λ*_2_=*λ*_1_^*∗*^, where *λ*_1_^*∗*^=(*r*/1 − *r*)*λ*_1_. First, we convert *X*_*i*_^*∗*^=(1 − *r*/*r*)*X*_*i*_, for *i*=1,…, *n*. Since *X*_*i*_^*∗*^ ~ *E*(*λ*_1_^*∗*^) we can perform $\left\{\begin{array}{c} H_{0}:R=r\\ K_{0}:R>r \end{array}\right.$, $\left\{\begin{array}{c} H_{0}:R=r\\ K_{1}:R<r \end{array}\right.$, and $\left\{\begin{array}{c} H_{0}:R=r\\ K_{2}:R\neq r \end{array}\right.$ based on independent samples *X*_1_^*∗*^,…, *X*_*n*_^*∗*^ and *Y*_1_,…, *Y*_*m*_. The corresponding hypothesis are $\left\{\begin{array}{c} H_{0}:\lambda_{2}=\lambda_{1}^{*}\\ K_{0}:\lambda_{2}>\lambda_{1}^{*} \end{array}\right.$, $\left\{\begin{array}{c} H_{0}:\lambda_{2}=\lambda_{1}^{*}\\ K_{1}:\lambda_{2}<\lambda_{1}^{*} \end{array}\right.$, and $\left\{\begin{array}{c} H_{0}:\lambda_{2}=\lambda_{1}^{*}\\ K_{2}:\lambda_{2}\neq \lambda_{1}^{*} \end{array}\right.$, by considering proof of Theorem 1 is completed.

For achieving an unbiased C.I for *R*, we check which one of values of *r* satisfies equations (15)–(17). These values construct mentioned C.I.

*Note 1*. Besides three basic tests $\left\{\begin{array}{c} H_{0}:R=r\\ K_{0}:R>r \end{array}\right.$, $\left\{\begin{array}{c} H_{0}:R=r\\ K_{1}:R<r \end{array}\right.$, and $\left\{\begin{array}{c} H_{0}:R=r\\ K_{2}:R\neq r \end{array}\right.$, one may wish to test hypotheses $\left\{\begin{array}{c} H_{3}:R\leq r_{1}\;or\;R\geq r_{2}\;\\ K_{3}:r_{1}<R<r_{2} \end{array}\right.$ and $\left\{\begin{array}{c} H_{4}:r_{1}\leq R\leq r_{2}\\ K_{4}:R<r_{1}\;or\;R>r_{2} \end{array}\right.$. However, these two hypotheses cannot be converted to tests based on *X*_ _^*∗*^ and *Y*. In other words, there is no linear function between *λ*_1_ and *λ*_2_ which is equivalent with hypotheses *H*_3_, *K*_3_, *H*_4_, and *K*_4_. Therefore, UMPUT does not exist for *H*_3_ against *K*_3_ and *H*_4_ versus *K*_4_.

## 3. Testing *R* in GL Distribution with Known and Common Scale Parameter

In this section, we compute asymptotic test and UMPUT of the stress-strength model for GL distribution. This is assumed that two-scale parameters are equal and known. An asymptotic test has been computed for comparison with UMPUT.

Suppose random variables *V* and *U* are independent and *V* ~ GL(*α*_1_, *λ*) and *U* ~ GL(*α*_2_, *λ*); then,

(18) $$\begin{array}{c} R=P\left(V>U\right)=\frac{\alpha_{1}}{\alpha_{1}+\alpha_{2}}. \end{array}$$

First, we find asymptotic test by using MLEs of parameters and their asymptotic distribution. Let *V*_1_,…, *V*_*m*_ ~ GL(*α*_1_, *λ*) and *U*_1_,…, *U*_*n*_ ~ GL(*α*_2_, *λ*); then, the likelihood function is

(19) $$\begin{array}{c} \iota =m\;\ln\left(\alpha_{1}\right)+\;n\;\ln\left(\alpha_{2}\right)+\left(m+n\right)\ln\left(\lambda \right)-\lambda \left(\sum_{i=1}^{m}v_{i}+\sum_{j=1}^{n}u_{j}\right)\\ -\left(\alpha_{1}+1\right)S_{1}\left(v,\lambda \right)-\left(\alpha_{2}+1\right)S_{1}\left(u,\lambda \right), \end{array}$$

where *S*_1_(*w*, *a*)=∑_*i*=1_^*q*^ln(1+*e*^−*a* *w*_*i*_^) and *w*=(*w*_1_,…, *w*_*q*_). By derivation with respect to parameters, we have

(20) $$\begin{array}{c} {\hat{\alpha }}_{1}=\frac{m}{S_{1}\left(V,\lambda \right)},{\hat{\alpha }}_{2}=\frac{n}{S_{1}\left(U,\lambda \right)}\;. \end{array}$$

By substitution $\hat{\eta }=\left({\hat{\alpha }}_{1},{\hat{\alpha }}_{2}\right)$ in equation (3), we have

(21) $$\begin{array}{c} \hat{R}=\frac{m\;S_{1}\left(U,\lambda \right)}{m\;S_{1}\left(U,\lambda \right)+n\;S_{1}\left(V,\lambda \right)}. \end{array}$$

Fisher's information matrix and its inverse are $J\left(\eta \right)=\left[\begin{array}{cc} \left(m/\alpha_{1}^{2}\right) & 0\\ 0 & \left(n/\alpha_{2}^{2}\right) \end{array}\right]$ and $J^{-1}\left(\eta \right)=\left[\begin{array}{cc} \left(\alpha_{1}^{2}/m\right) & 0\\ 0 & \left(\alpha_{2}^{2}/n\right) \end{array}\right]$.

Since $\hat{\eta }⟶N_{2}\left(\eta ,J^{-1}\left(\eta \right)\right)$, by the multivariate Delta method, one can see that ${\hat{R}}_{\;}⟶N\left(R,\sigma^{2}\left(\eta \right)\right)$, where *σ*^2^(*η*)=(∂*R*^*G*^/∂*η*)*J*^−1^(*η*)(∂*R*^*G*^/∂*η*)^*T*^, (∂*R*^*G*^/∂*η*)=((∂*R*^*G*^/∂*α*_1_), (∂*R*_ _^ *G*^/∂*α*_2_)), (∂*R*^*G*^/∂*α*_1_)=(*α*_2_/(*α*_1_+*α*_2_)^2^), and (∂*R*^*G*^/∂*α*_2_)=(−*α*_1_/(*α*_1_+*α*_2_)^2^). Therefore, $\left(\hat{R}-R/{\hat{\sigma }}_{\text{GL}}\right)⟶N\left(0,1\right)$ as *n* and *m* tend to infinity and (*n*/*m*)⟶*d*, for a 0 < *d* < +*∞*, where

(22) $$\begin{array}{c} {\hat{\sigma }}_{\text{GL}}^{2}=\frac{n+m}{nm}\frac{{\hat{\alpha }}_{1}^{2}{\hat{\alpha }}_{2}^{2}}{{\left({\hat{\alpha }}_{1}+{\hat{\alpha }}_{2}\right)}^{4}}. \end{array}$$

Now, the (1 − *α*) × 100 percentage C.I of *R* and its corresponding tests based on normal approximation for hypotheses *K*_0_, *K*_1_, and *K*_2_ are the same with Section 2 with ${\hat{\sigma }}_{\text{GL}}^{2}$ in equation (22) instead of ${\hat{\sigma }}_{1}^{2}$.

### 3.1. UMPUT for *R* in GL Distribution

For UMPUT of *K*_0_, *K*_1_, and *K*_2_, we only need to convert original data to *X*_*i*_=ln(1+*e*^−*λ* *U*_*i*_^), for *i*=1,…, *n*, and *Y*_*j*_=ln(1+*e*^−*λ* *V*_*j*_^), for *j*=1,…, *m*. This can be simply seen as *X*_1_,…, *X*_*n*_ ~ *E*(*α*_2_) and *Y*_1_,…, *Y*_*m*_ ~ *E*(*α*_1_). From this point, all results of Theorems 1 and 2 can be used here with substitution *α*_2_ and *α*_1_ instead *λ*_1_ and *λ*_2_. To put it more clearly, first of all, we define three statistics *U*_GL_=((1 − *r*)*S*_1_(*U*, *λ*)/(1 − *r*)*S*_1_(*U*, *λ*)+*rS*_1_(*V*, *λ*)), *S*_GL_^*∗*^=(1 − *r*/*r*)*S*_1_(*U*, *λ*), and *T*_GL_^*∗*^=(1 − *r*/*r*)*S*_1_(*U*, *λ*)+*S*_1_(*V*, *λ*). Now, CRs of UMPUTs for *K*_0_ and *K*_1_ are, respectively,

(23) $$\begin{array}{c} \text{C}{\text{.R}}_{\text{UMPU},0}=\left\{U_{GL}>q\text{beta}\left(1-\alpha ,n,m\right)\right\} \end{array}$$

and

(24) $$\begin{array}{c} \text{C}{\text{.R}}_{\text{UMPU},1}=\left\{U_{\text{GL}}<q\text{beta}\left(\alpha ,n,m\right)\right\}. \end{array}$$

Also, the acceptance region of UMPUT for hypothesis *K*_2_ is achieved from the following equation:

(25) $$\begin{array}{c} \text{A}{\text{.R}}_{\text{UMPU},2}=\left\{c_{1}\left(\alpha ,T_{\text{GL}}^{*}\right)<S_{\text{GL}}^{*}<c_{2}\left(\alpha ,T_{\text{GL}}^{*}\right)\right\}, \end{array}$$

where two values *c*_1_(*α*, *T*_GL_^*∗*^) and *c*_2_(*α*, *T*_GL_^*∗*^) are determined by the following equations:

(26) $$\begin{array}{c} p\text{beta}\left(\frac{c_{2}\left(\alpha ,T_{\text{GL}}^{*}\right)}{T_{\text{GL}}^{*}},n,m\right)-p\text{beta}\left(\frac{c_{1}\left(\alpha ,T_{\text{GL}}^{*}\right)}{T_{\text{GL}}^{*}},n,m\right)=1-\alpha , \end{array}$$

(27) $$\begin{array}{c} p\text{beta}\left(\frac{c_{2}\left(\alpha ,T_{\text{GL}}^{*}\right)}{T_{\text{GL}}^{*}},n+1,m\right)-p\text{beta}\left(\frac{c_{2}\left(\alpha ,T_{\text{GL}}^{*}\right)}{T_{\text{GL}}^{*}},n+1,m\right) \end{array}$$

Again similar with the previous section, for getting an unbiased C.I. for *R* in case of GL model, we check which one of the values of *r* satisfies equations (25)–(27). Then, one can have an unbiased C.I. Also, Note 1 remains right here in case of GL distribution.

## 4. Comparison between UMPUT and Asymptotic Test

In this section, we compare two tests for hypotheses *K*_0_, *K*_1_, and *K*_2_ based on UMPUT and asymptotic test. First, we show tests done by asymptotic distribution of $\hat{R}$ are unbiased for three mentioned hypotheses.

**Lemma 1 .**

Let *X*_1_,…, *X*_*n*_ ~ *E*(*λ*_1_) and *Y*_1_,…, *Y*_*m*_ ~ *E*(*λ*_2_) and C.R*s* for testing hypotheses *K*_0_, *K*_1_, and *K*_2_ are C.R_*as*,0_, C.R_*as*,1_, and C.R_*as*,2_. Then, these tests are unbiased.

**Proof**

We prove theorem for three hypotheses separately by computation of its power function for a fixed point *r*_1_ in alternative hypothesis *β*(*r*_1_)=*P*_*R*=*r*_1__(C.R). For hypothesis *K*_0_ : *R* > *r*, C.R is C.R_*as*,0_={*Z* > *z*_1−*α*_} for $Z=\left(\hat{R}-r/{\hat{\sigma }}_{1}\right)$. So, the power function is

(28) $$\begin{array}{c} \beta \left(r_{1}\right)=P_{R=r_{1}}\left(\frac{\hat{R}-r}{{\hat{\sigma }}_{1}}>z_{1-\alpha }\right)=P\left(Z>z_{1-\alpha }-\frac{r_{1}-r}{\sigma_{1}}\right)=1-\Phi \left(z_{1-\alpha }-\frac{r_{1}-r}{\sigma_{1}}\right). \end{array}$$

Since *r*_1_ > *r*, we have *β*(*r*_1_) > *α*. For hypothesis *K*_1_ : *R* < *r*, similar to the previous case, we have *β*(*r*_1_)=Φ(*z*_*α*_ − (*r*_1_ − *r*/*σ*_1_)) which for *r*_1_ < *r* is clearly bigger than *α*. For the last case, the proof is complicated. For *K*_2_ : *R* ≠ *r*, one can show *β*(*r*_1_)=*P*(*Z* > *d*_1_)+*P*(*Z* < *d*_2_), where *d*_1_=*z*_1−(*α*/2)_ − (*r*_1_ − *r*/*σ*_1_) and *d*_2_=−*z*_1−(*α*/2)_ − (*r*_1_ − *r*/*σ*_1_). Now, we consider two cases *r*_1_ > *r* and *r*_1_ < *r* separately. If *r*_1_ > *r*, we have

(29) $$\begin{array}{c} \beta \left(r_{1}\right)=P\left(Z>z_{1-\left(\alpha /2\right)}\right)+P\left(Z<-z_{1-\left(\alpha /2\right)}\right)+P\left(d_{1}<Z<z_{1-\left(\alpha /2\right)}\right)-P\left(d_{2}<Z<-z_{1-\left(\alpha /2\right)}\right). \end{array}$$

By these facts that two intervals (*d*_1_, *z*_1−(*α*/2)_) and (*d*_2_, −*z*_1−(*α*/2)_) have equal lengths and second interval is placed in tail of Standard Normal distribution, it has less probability than the first interval. This demonstrates that *β*(*r*_1_) > *α*. For case of *r*_1_ < *r*, the power function is as follows:

(30) $$\begin{array}{c} \beta \left(r_{1}\right)=P\left(Z>z_{1-\left(\alpha /2\right)}\right)+P\left(Z<-z_{1-\left(\alpha /2\right)}\right)+P\left(-z_{1-\left(\alpha /2\right)}<Z<d_{2}\right)-P\left(z_{1-\left(\alpha /2\right)}<Z<d_{1}\right). \end{array}$$

By similar logic as case of *r*_1_ > *r*, one can show that *β*(*r*_1_) > *α*. These results complete the proof.

Now, we can compare two tests, asymptotic and UMPUT, in the following theorem.

**Theorem 3 .**

Let *X*_1_,…, *X*_*n*_ ~ *E*(*λ*_1_) and *Y*_1_,…, *Y*_*m*_ ~ *E*(*λ*_2_) and asymptotic C.R*s* for testing hypotheses *K*_0_, *K*_1_, and *K*_2_ be C.R_*as*,0_, C.R_*as*,1_, and C.R_*as*,2_. Then, UMPUTs based on equations (13)–(17) are more powerful than these asymptotic tests.

**Proof**

The proof comes from unbiasedness of asymptotic tests in Lemma 1 and definition of UMPUT.

Theorem 3 guaranties that the unbiased C.I earned by using of acceptance region of *K*_2_ is more accurate than asymptotic C.I.

All results achieved about comparison between asymptotic test and UMPUT for exponential distribution are satisfied for GL distribution, too. In the other words, Lemma 1 and Theorem 3 are held for case of GL distributed components. The most important matter which has value for repeat is this point that UMPUTs based on equations (23)–(27) are more powerful than the asymptotic tests by using of statistic $Z=\left(\hat{R}-r/{\hat{\sigma }}_{\text{GL}}\right)$.

## 5. Conclusion and Future Works

In this paper, we found UMPUT for stress-strength quantity in case of exponential and GL distributed components, respectively. By using this test in two sides' case, C.I for *R* was achieved. This has been proved that UMPUT is more powerful than the asymptotic test. Our methodology has been used on stress-strength models for the first time.

As we mentioned in Section 1, a numerous distributions have been applied to estimation of stress-strength quantity. In almost all of these papers, estimation is performed by MLE and its consistency property. Also, in some cases the Bayesian estimation is performed. These methods have an appropriate performance usually for large data sample size. The methodology introduced in this article can be applied to other distributions such as generalized exponential, generalized Pareto, Kumaraswamy, etc. (see Alshanbari et al. [14]).

Saber and Yousof [15] surveyed a generalization of stress-strength models named generalized stress-strength models (*R*^*G*^),

(31) $$\begin{array}{c} R^{G}=P\left(Y<\;X<Z\right), \end{array}$$

for GL distribution. Finding UMPUT for testing this quantity is an interesting work which may be done in future.

Recently, the study of *R* by censoring data has been expanded by many authors. For instance, Abu-Moussa et al. [16] and Almongy et al. [17] studied *R* under progressive censoring data for Rayleigh and Weibull extended distributed components, respectively. The study on UMPUT of *R* for censoring data can be a challenging and interesting work for the future.

## Data Availability

No data were used to support the findings of the study.
